# Evaluation of the prognostic value of lncRNA UCA1 combined with extravascular lung water index and lung ultrasound score in patients with acute lung injury

**DOI:** 10.1111/crj.13651

**Published:** 2023-06-15

**Authors:** Zhaopeng Jiang, Jiaqi Wu, Ming Wan, Lingling Liu, Xianli Zhou

**Affiliations:** ^1^ Department of In‐Patient Ultrasound The 2nd Affiliated Hospital of Harbin Medical University Harbin China; ^2^ Medical Record Office The 2nd Affiliated Hospital of Harbin Medical University Harbin China

**Keywords:** acute lung injury, EVLWI, LUS, prediction, UCA1

## Abstract

**Introduction:**

Acute lung injury (ALI) is a common and rapidly developing critical inflammatory lung disease in clinic. This study investigated the predictive value of lncRNA UCA1, extravascular lung water index (EVLWI), and lung ultrasound score (LUS) in predicting the overall outcome of patients with ALI.

**Methods:**

Patients with ALI were recruited for detecting the content of UCA1, EVLWI, and LUS. All patients were cataloged into the survival group and death group according to the prognosis. The discrepancy of UCA1, EVLWI, and LUS was compared in the two groups. The prognostic significance of UCA1, EVLWI, LUS, and their combination was estimated by logistic regression and the receiver operating characteristic (ROC) curve.

**Results:**

The levels of UCA1, LUS, and EVLWI were elevated in the death group compared with the survival group. The content of UCA1 was positively correlated with LUS scores and EVLWI scores. UCA1, LUS, and EVLWI were independent indicators of predicting the prognosis of patients with ALI. The ROC curve reflected that UCA1, LUS, and EVLWI could forecast the endpoint events of patients with ALI whereas their combined approach had the highest accuracy.

**Conclusion:**

Highly expressed UCA1 is a biomarker in forecasting the outcome of patients with ALI. It had high accuracy in predicting the endpoint of patients with ALI when combined with LUS and EVLWI.

## Graphical abstract

Patients with ALI were cataloged into the survival group and death group according to the prognosis. The relative expression of lncRNA UCA1, the scores of EVLWI, and the LUS score were detected in patients with ALI. The ROC curve and the logistic regression analysis showed that highly expressed UCA1 was a biomarker in forecasting the outcome of patients with ALI. It had high accuracy in predicting the endpoint of patients with ALI when combined with LUS and EVLWI.

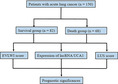

## 1 INTRODUCTION

Acute lung injury (ALI) is a pulmonary lesion caused by direct and indirect factors, thus leading to tachypnea and hypoxia.^1^ As a lower respiratory infection, ALI is mainly triggered by various bacteria, viruses, and fungal or parasitic infections.^2^ Beyond these reasons, serious infection, trauma, massive transfusion, and drowning are crucial elements that stimulate ALI.^3^ The management of ALI included control of inflammatory responses, respiratory support, and liquid supplements. However, mortality and long pulmonary damage are still at a high level. Persistent and uncontrolled ALI may elicit respiratory distress syndrome (RDS), causing shock, ischemia, and hypoxia.^4^ Therefore, prompt diagnosis of ALI is a premise for better survival.

LncRNA is a kind of non‐coding RNA that is widely researched in several illnesses. The clinical significance of lncRNAs is currently disclosed by researchers.^5^ LncRNA UCA1 is one hotpot RNA in the study of human disorders. In the SiO_2_ dust‐treated mouse silicosis model, the expression of UCA1 is correlated to the presence of silicosis.^6^ As documented by Chen et al., UCA1 is highly expressed in patients with acute RDS and has the clinical possibility of differentiating these kinds of patients.^7^ Collectively, UCA1 may be an abnormally expressed lncRNA in ALI, and its screening significance catches our attention. Extravascular lung water index (EVLWI) is a common parameter in screening pulmonary edema. In patients with lung injury, the EVLWI is elevated, and its increase is related to the aggressive respiratory index, and it improves the clinical diagnostic value of ALI, acute RDS, and severe lung injury.^8^ Letourneau et al reported that EVLWI is a diagnostic marker in patients with ALI because of its increased levels.^9^ Pulmonary ultrasound has become a routine examination of various lung diseases.^10^ Lung ultrasound can evaluate pleural effusion, pneumothorax, and changes in lung parenchyma, and it is an important way to diagnose and predict lung lesions.^11^ Thus, these arouse our interest.

Herein, 150 patients with ALI were volunteers in this research for the purpose of detecting the clinical roles of the combination of UCA1, lung ultrasound score (LUS), and EVLWI. According to the outcomes of each patient, all patients were cataloged into the survival group and death group. The expression of UCA1 was assessed in patients with ALI and the discrepancy of its levels in survivors and nonsurvivors was computed. The relationships were disclosed between UCA1 and LUS score and EVLWI levels. The predictive likelihood of all included items was revealed by logistic correlation analysis. Additionally, the receiver operating characteristic (ROC) curves were plotted to estimate the value of the single index and joint diagnosis.

## 2 MATERIALS AND METHODS

### 2.1 Volunteers and grouping

Totally, 150 patients diagnosed with ALI between June 2019 and July 2022 were recruited in the present research. Patients were verified through clinical characteristics, arterial blood gas analysis, analysis of pathogenic factors, and radiographic information based on the Berlin definition of acute RDS (ARDS).^12^ The specific standards were as follows: (1) an acute onset of dyspnea with a history of aspiration within 1 week and incubation for mechanical ventilation support; (2) oxygenation index (arterial oxygen/fraction of inspired oxygen) ≤300 mmHg; (3) new infiltration shadow on chest imaging. All patients were onset within 24 h and volunteered for this study with submission of written approval consent. Individuals with malignancies, pregnancy, history of chronic obstructive pulmonary disease, pulmonary embolism, asthma, and other respiratory diseases, interstitial lung disease, autoimmune diseases, and incomplete medical records could not cooperate with this study.^13^ Included patients were cataloged into the survival group and death group as per their outcome in the duration of the hospital. The approval of this study was obtained from the ethics committee of the 2nd Affiliated Hospital of Harbin Medical University. All procedures performed in studies involving human participants were in accordance with the Declaration of Helsinki.

### 2.2 EVLWI and LUS obtain

**FIGURE 1:**
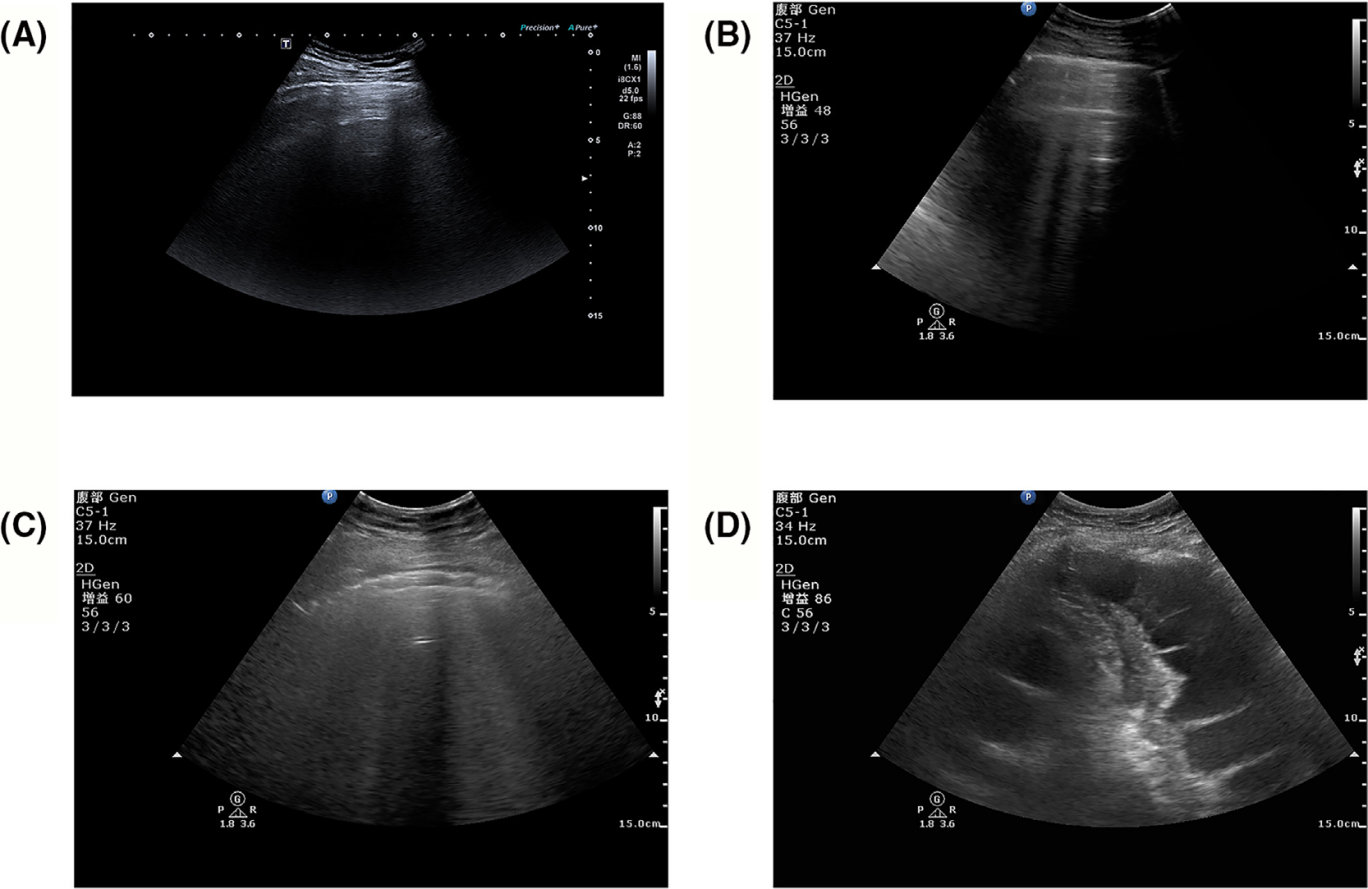
Four types of aeration ultrasound patterns. (A) Presence of A line (scored as 0). (B) Presence of B lines (scored as 1). (C) Presence of coalescent B lines (scored as 2). (D) Presence of lung consolidation (scored as 3).

The clinical parameters (EVLWI and LUS) were both gained on the first day of hospitalization. Patients undergo strict evaluation of indications and contraindications before using pulse index continuous cardiac output (PiCCO). ALI patients were monitored with the PiCCO technique (Pulsion Medical System, Munich, Germany), and the EVLWI levels were derived from this system using single indicator transpulmonary thermodilution measurement. Regarding LUS, the chest wall of each patient was divided into 12 areas. The LUS scores were the sum of estimated values for each site. Each district was graded according to the following signs: A lines or less than two isolated B lines marked as 0, discrete B lines marked as 1, fused B lines marked as 2, and lung consolidation area marked as 3.^14^ The representative pictures were shown in Figure 1. The higher scores indicated smaller lung damage.

### 2.3 UCA1 expression assessment

The blood specimens were extracted from patients within 24 h of hospitalization and the plasma was then collected and deposited at −80°C. Trizol reagent (Invitrogen, America) was used for the extraction of total RNA. RevertAid first strand of complementary DNA (cDNA) synthesis kit (Thermo Scientific, America) was utilized with total RNA for synthesizing cDNA. The relative expression of UCA1 was estimated using an lncRNA qPCR kit (SYBR Green) from Abnova (Shanghai, China) on ABI 7700 Sequence Detector (Applied Biosystems, America). The reference gene was GAPDH. The expression was normalized using the 2^−∆∆CT^ formula. The  following sequences were the primers: UCA1, upstream, 5′‐GCCAGCCTCAGCTTAATCCA‐3′ and downstream, 5′‐CCCTGTTGCTAAGCCGATGA‐3′; GAPDH upstream, 5′‐ACCCACATCCCTCAGACAC‐3′ and downstream, 5′‐CCCCAATACGACCAAATCC‐3′.

### 2.4 Statistical analysis

The comparisons between groups were calculated using the independent *T*‐test and *χ* ^2^ method. The interrelationships between UCA1 and EVLWI and LUS scores were estimated using Pearson correlation method. The ROC curve, logistic regression method, and forest plots were depicted to reveal the predictive significance of UCA1, EVLWI, and LUS levels. Statistical significance was reflected by *p* < 0.05.

## 3 RESULTS

### 3.1 Clinicopathological information of patients

In the present study, all patients were transferred to the intensive care unit (ICU). The average length of stay in hospital was 18.15 ± 4.78 days, and the average length of ICU stay was 12.65 ± 2.68 days. Twenty‐seven ALI patients suffered from heart diseases and had respiratory failures not fully explained by cardiac failure. In line with the prognosis of each patient, all included patients with ALI were divided into the survival group (*n* = 82) and death group (*n* = 68). The baseline information of patients with ALI was compared between the survival group and the death group. The age, gender distribution, and body mass index had no distinction between the survival group and death group (*p* > 0.05, Table 1). Two basic diseases (diabetes and hypertension), as well as respiration rat and heart rate, had no undifferentiated result between the two groups (*p* > 0.05, Table 1). The LUS and EVLWI scores were increased in the death group compared with the survival group (*p* < 0.001, Table 1).

**TABLE 1:** Basic information statistics of patients.

Items	Survival (*n* = 82)	Death (*n* = 68)	Significant (*p*)
Gender (male/female)	39/43	28/40	0.434
Age (years)	59.15 ± 9.01	58.06 ± 8.52	0.452
BMI (kg/m^2^)	23.12 ± 2.29	23.49 ± 1.77	0.280
History of diabetes (*n*/%)	13/15.85	8/11.76	0.472
History of hypertension (*n*/%)	18/21.95	19/27.94	0.397
Respiration rate (times/min)	29.37 ± 5.07	30.75 ± 4.56	0.084
Heart rate (times/min)	106.26 ± 19.70	102.01 ± 19.31	0.187
LUS score	16.37 ± 4.59	22.06 ± 4.37	<0.001
EVLWI score	9.9 ± 1.79	15.07 ± 4.51	<0.001

*Note*: Data were expressed mean ± standard deviation or *n*.

Abbreviations: BMI, body mass index; EVLWI, extravascular lung water index; LUS, lung ultrasound score.

### 3.2 Discrepancy of UCA1 expression between the two groups

**FIGURE 2:**
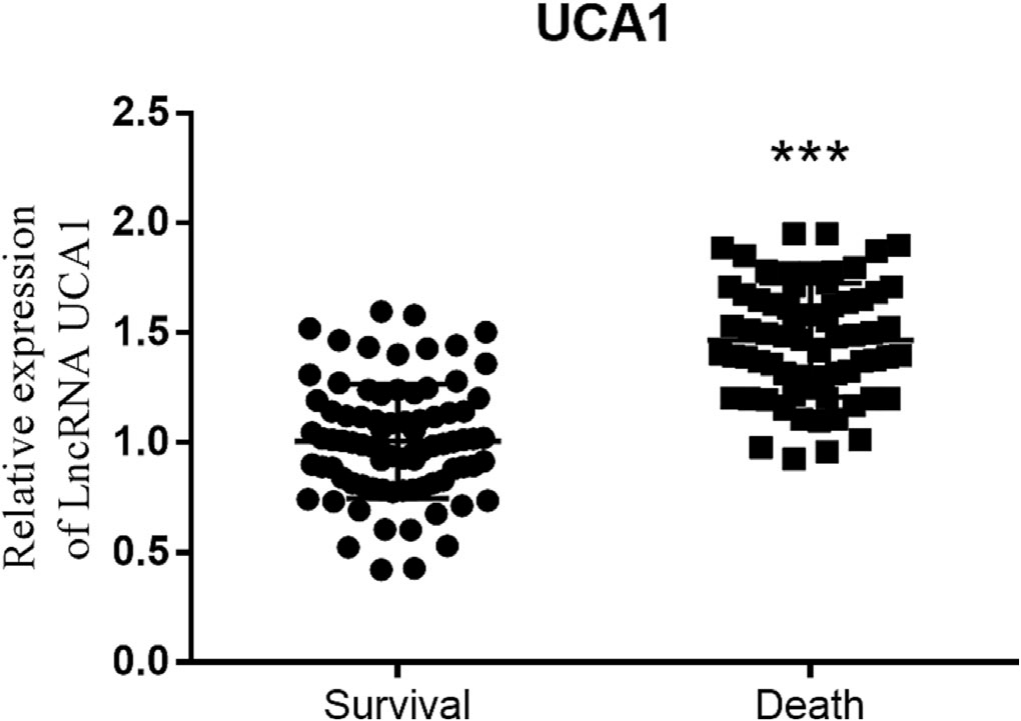
The content of UCA1 in survival group and death group. ***p < 0.001, relative to survival group.

The content of plasma UCA1 expression in patients with ALI was assessed. Patients with death diagnosis had a higher level of UCA1 expression than survival group, reflecting that the aggressive ALI progression might disturb the normal expression of UCA1 (*p* < 0.001, Figure 2).

### 3.3 UCA1 related to EVLWI and LUS

**FIGURE 3:**
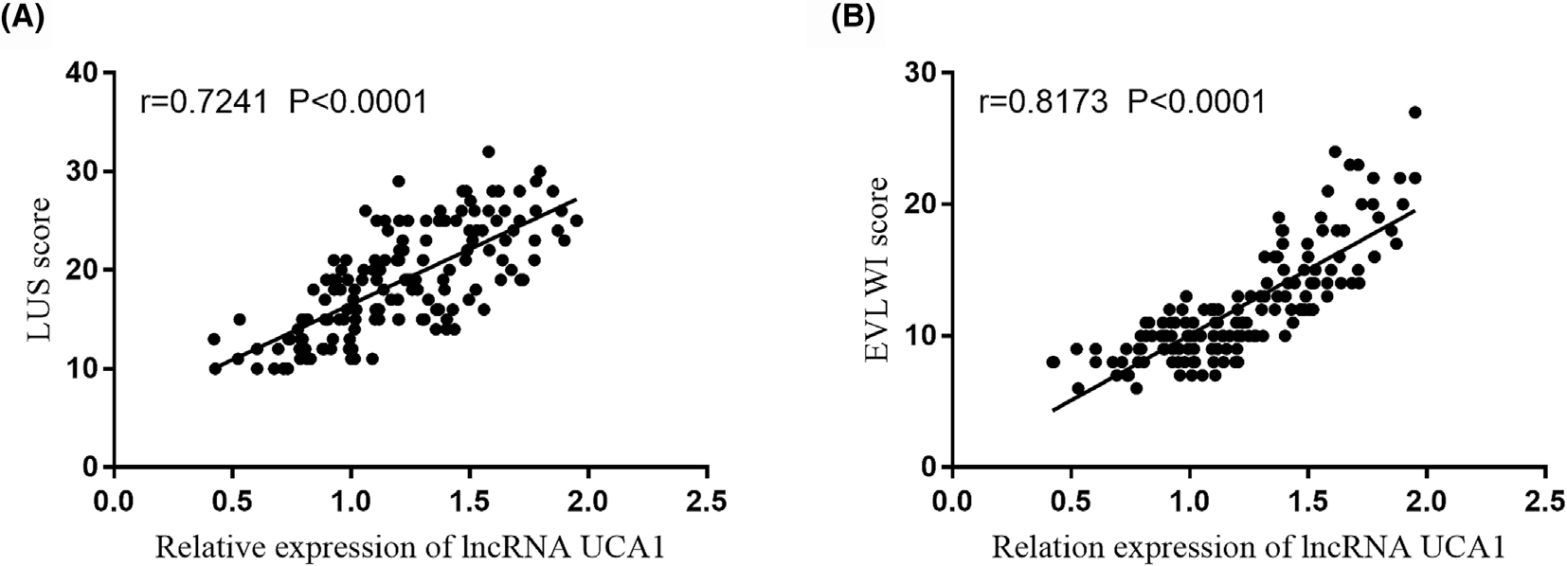
UCA1 was related to (A) lung ultrasound score (LUS) and (B) extravascular lung water index (EVLWI).

The Pearson correlation method was applied to describe the interconnections of UCA1 with LUS and EVLWI. The concentration of UCA1 was positively correlated with LUS (*r* = 0.724, *p* < 0.001, Figure 3A). The overexpression of UCA1 was associated with the increase of EVLWI scores in patients with ALI (*r* = 0.817, *p* < 0.001, Figure 3B). These results documented that the alternation of plasma UCA1 may relate to the situation of extravascular lung water, thus reflecting the lung damage.

### 3.4 Predictive performance

**FIGURE 4:**
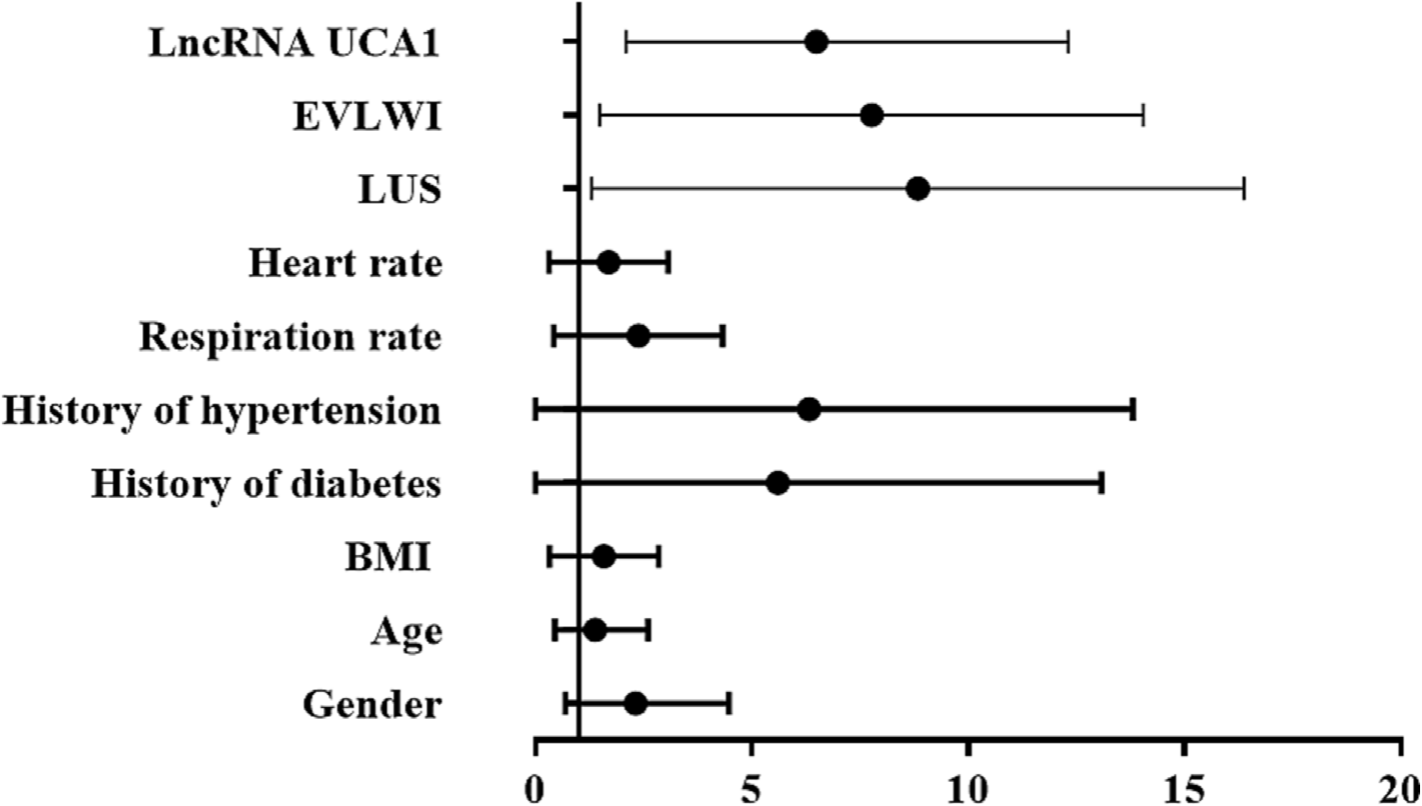
Forest plot of logistic regression analysis.

The logistic regression analysis was the method for evaluating the correlations of parameters with prognostic status. As depicted in Table 2, LUS (odds ratio [OR] = 6.699, 95% confidence interval [CI] [2.607, 17.216], *p* < 0.001), EVLWI (OR = 6.080, 95% CI [2.512, 14.718], *p* < 0.001), and UCA1 (OR = 5.078, 95% CI [2.094, 12.315], *p* < 0.001) were independent indicators for forecasting the outcome of patients with ALI. The OR value and 95% CI value of each item were clearly drawn in the forest plot (Figure 4).

**TABLE 2:** Correlation of each index with prognostic status.

Items	OR	95% CI	Significant (*p*)
Gender	1.768	[0.699, 4.468]	0.228
Age	1.084	[0.452, 2.600]	0.857
BMI	1.254	[0.528, 2.978]	0.608
History of diabetes	2.280	[0.367, 14.169]	0.376
History of hypertension	3.386	[0.773, 14.830]	0.106
Respiration rate	1.848	[0.751, 4.544]	0.181
Heart rate	1.316	[0.538, 3.218]	0.548
LUS	6.699	[2.607, 17.216]	<0.001
EVLWI	6.080	[2.512, 14.718]	<0.001
LncRNA UCA1	5.078	[2.094, 12.315]	<0.001

Abbreviations: BMI, body mass index; CI, confidence interval; EVLWI, extravascular lung water index; LUS, lung ultrasound score; OR, odds ratio.

**FIGURE 5:**
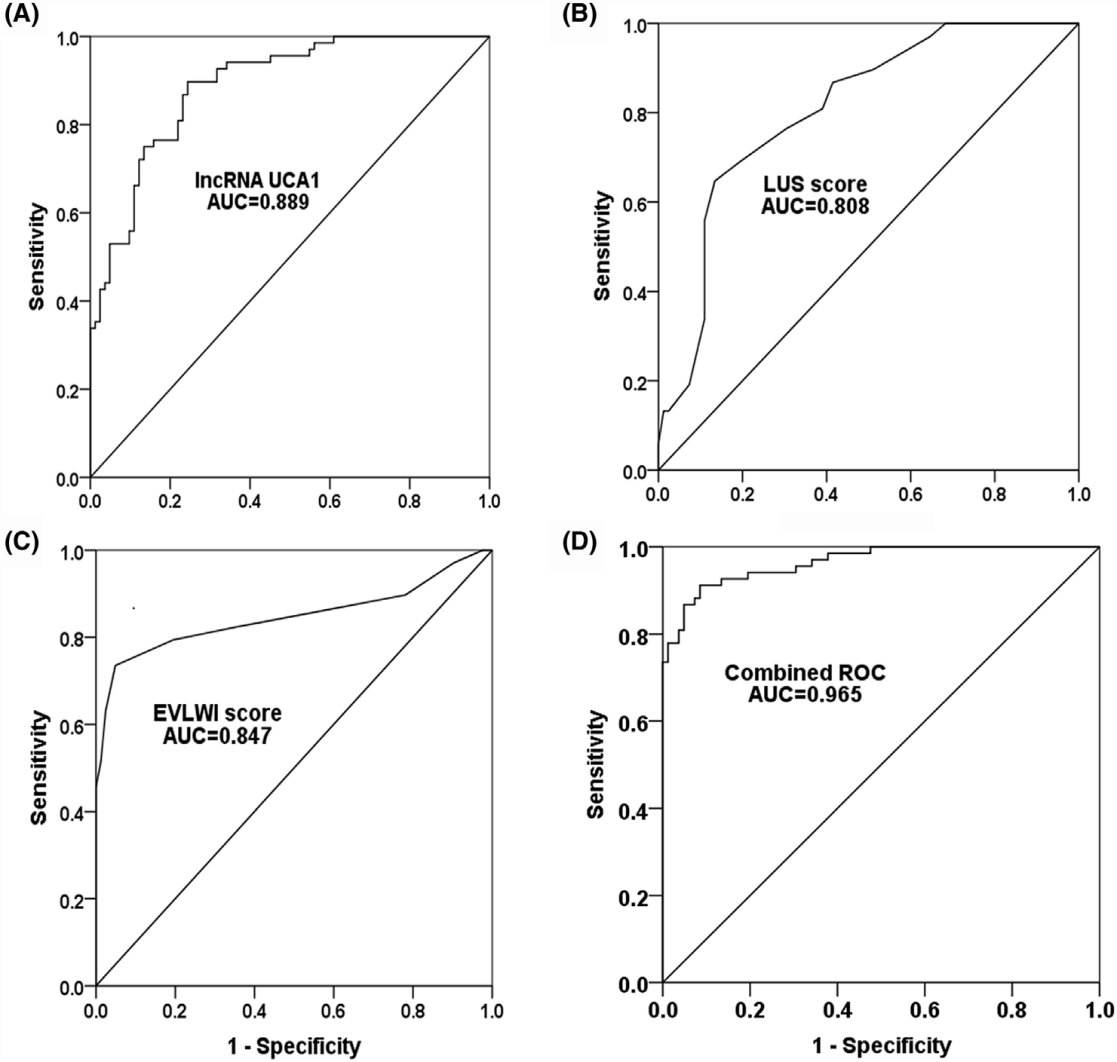
Receiver operating characteristic (ROC) curve of plasma (A) UCA1, (B) lung ultrasound score (LUS), (C) extravascular lung water index (EVLWI), and (D) their combination to predict the death of acute lung injury (ALI) patients.

The ROC curves were also plotted to analyze the predictive potential of UCA1, LUS, and EVLWI. As documented in Figure 5A, the expression of plasma UCA1 had a certain possibility of being used as a biomarker for ALI (AUC = 0.889). The pictures of Figure 5B,C show the clinical significance of LUS and EVLWI in diagnosing the final outcomes of patients with ALI (AUC = 0.808 and AUC = 0.847, respectively). Although these three indicators had a certain value in distinguishing the survival or death outcome of patients, they had a relatively low specificity or sensitivity (Table 3). Therefore, the combined diagnosis was analyzed using the ROC curve. The finding verified that the combined UCA–LUS–EVLWI approach had high accuracy in screening the prognosis of patients with ALI (AUC = 0.965, Figure 5D and Table 3).

**TABLE 3:** Basic information statistics of ROC curve.

Items	LncRNA UCA1	LUS	EVLWI	Combined ROC
AUC	0.889	0.808	0.847	0.965
Standard error	0.026	0.035	0.036	0.013
95% CI	[0.839, 0.939]	[0.738, 0.877]	[0.776, 0.919]	[0.940, 0.990]
Sensitivity	89.7%	64.7%	73.5%	91.2%
Specificity	75.6%	86.6%	95.1%	91.5%
*p* value	<0.001	<0.001	<0.001	<0.001

Abbreviations: CI, confidence interval; EVLWI, extravascular lung water index; LUS, lung ultrasound score; ROC, receiver operating characteristic.

## 4 DISCUSSION

ALI is a kind of lung infection injury caused by acute inflammation, and it is a commonly severe respiratory disease in intensive care units.^15^ When severe lung injury is not timely and effectively controlled, it may aggravate lung tissue edema, further impair lung function, and eventually develop into acute RDS, with an overall mortality rate as high as 25%–45%.^16^ At present, there is still a lack of effective treatment for ALI in clinic, thus causing serious complications and adverse outcomes.^17^ However, the existing chest physical examination lacks specificity in ALI progression. Therefore, how to predict the severity and prognosis of patients in early stage is of great significance to improve the prognosis of patients.

LncRNA is commonly researched in human disorders, such as diseases of the respiratory system. LncRNA CASC2 is down‐regulated, lncRNA MALAT1 is up‐regulated, and lncRNA MIR3142HG is overexpressed in patients with ALI, indicating that their expression may be influenced by the occurrence of ALI.^18^, ^19^, ^20^ The content of UCA1 is elevated and related to the disease severity and prognosis in patients with sepsis, which may become the cause of ALI.^21^ In the pneumonia models constructed using ligation and puncture and lipopolysaccharide, the levels of UCA1 are increased, and it can aggravate the development of pneumonia.^22^ In the present research, the expression of UCA1 was elevated in the ALI patients with death diagnosis, which suggested that UCA1 may be involved in the progression of ALI. This finding of UCA1 expression was consistent with the aforementioned results. Many lncRNAs are implicated in pulmonary disorders and they may serve as indicators of progression of diseases. In lung adenocarcinoma and non‐small cell lung cancer, the overexpression of UCA1 indicates a poor prognosis for patients.^23^, ^24^ LncRNA ELFN1‐AS1 is a predictor of poor survival of patients with non‐small cell lung cancer.^25^ The expression of RP5‐977B1 is proven to predict the diagnosis and prognosis of patients with non‐small cell lung cancer.^26^ However, the papers on the diagnosis of ALI are few. This investigation used the expression of UCA1 in ALI patients with different prognoses to reveal the predictive potential of UCA1 with the progression of ALI. The logistic regression analysis and ROC curve both reflected that UCA1 is an independent biomarker in predicting the prognosis of patients with ALI. The high expression of UCA1 indicated a poor outcome in patients with ALI.

Lung ultrasound is an important noninvasive method to evaluate the ratio of water to gas in lung and reflect lung injury, like pneumonia and lung consolidation.^27^ Liu et al. reported that lung ultrasound monitoring on patients with lung lesions can decrease the overall death rate and poor prognosis, suggesting that this tool can reflect the progression of lung disease.^28^ Li et al. document that LUS combined with other three indicators had high efficacy in predicting the prognosis of ALI patients.^29^ The combination of miR‐21‐3p and LUS is involved in the aggression and overall outcome of patients with ALI, reflecting that the expression of miRNA may correlate with LUS in ALI.^30^ The expression of UCA1 is elevated in patients with ARDS, and knockdown of UCA1 can reduce lung injury through inhibiting inflammatory response, reflecting that UCA1 is involved in ALI.^31^ In the current research, the expression of UCA1 was positively related to the LUS scores in patients with ALI, reflecting that UCA1 is associated with the severity of lung injury. In addition, LUS score could distinguish ALI patients with death prognosis from survival ones. EVLWI is another index commonly applied to monitor patients with ALI. The clinical significance of EVLWI in lung diseases has been documented by several reporters. EVLWI predicts the severity and development of ALI, and its prediction is prior to the criteria of ALI.^9^ Another research by Craig et al. reveals that the measurement of high EVLWI indicates the death risk of patients with ALI.^32^ This paper investigated the levels of EVLWI and analyzed the relationship between EVLWI and UCA1. We unveiled that the concentration of UCA1 was associated with EVLWI, indicating UCA1 is a risk factor for ALI. The detection of EVLWI could differentiate patients with poor prognosis from those with survival prognosis. The predictive potential of the combination of UCA1, LUS, and EVLWI was disclosed, and the finding found that their combination had high accuracy in forecasting the prognosis of patients with ALI. However, this study is a preliminary exploration study, which aimed to provide a novel thought for the study of clinical biomarkers. Additional large‐scale prospective studies are required to verify our findings. Having no comparison of the discrepancy between infectious and non‐infectious diseases as causes of ALI is a limitation of this retrospective study.

To sum up, the level of plasma UCA1 was elevated in ALI patients with death prognosis. The content of UCA1 was closely related to EVLWI and LUS scores. The combination of UCA1, LUS, and EVLWI had certain value for the prognosis evaluation of ALI patients, providing a new idea for understanding the pathogenesis and targeted therapy of ALI.

## AUTHOR CONTRIBUTIONS

Zhaopeng Jiang and Xianli Zhou designed the research study. Jiaqi Wu, Ming Wan, and Lingling Liu performed the research. Zhaopeng Jiang and Xianli Zhou analyzed the data. Zhaopeng Jiang wrote the manuscript. All authors contributed to editorial changes in the manuscript. All authors read and approved the final manuscript.

## CONFLICT OF INTEREST STATEMENT

There is no conflict of interest in this study.

## ETHICS STATEMENT

The approval of this study was obtained from the ethics committee of the 2nd Affiliated Hospital of Harbin Medical University. All procedures performed in studies involving human participants were in accordance with the Declaration of Helsinki.

## PATIENT CONSENT STATEMENT

All patients were volunteered for this study with submission of written approval consent.

## Data Availability

The data that support the findings of this study are available from the corresponding author upon reasonable request.
